# Event and Comment

**Published:** 1920-10

**Authors:** 


### DENTAL REGISTER

THE MAGAZINE OF DENTISTRY

Vol. LXXIV OCTOBER, 1920 No. 10

### EVENT AND COMMENT

**National Dental Association** The twenty-fourth annual session was held in Boston, August 23rd to 27th. It was the largest attendance ever registered, 6,612; although the association lists now about 27,000 members. The secretary's report shows gross assets of nearly $300,000 in seven years. There is more than $50,000 now in the National Relief Fund, and it is hoped that the $100,000 which is needed to make active distribution of grants will soon be secured. It is hoped that a large endowment for the literary and research work may be secured during the next five years, and that greater stability in the development of the profession may thereby be encouraged and assured. Dr. H. E. Friesell, of Pittsburg, was elected president, and Dr. T. B. Hartzell, of Minneapolis, was made president-elect. Milwaukee was chosen as the place of meeting for the 1921 session, and it is proposed to make it the "silver anniversary session."

The weather was favorable and the accommodations for the crowd was all that was expected from this admirable place for a meeting. The only criticism we heard was that the attendants could not see or hear all the good things that the program had to offer; but this is the usual complaint and no one as yet has been wise enough to overcome this objection to a large meeting. The special sections have helped some.

**The Necessity for Balance in Practice** It should not be difficult to establish order or system in most technical procedures, because habit is such a constant and controlling factor in most of the things we learn and do, by routine methods at any rate. Ordinarily, the practice of the routine or habitual makes for conviction and the establishment of better procedures, unless we are given to careless or freakish habits, to say nothing of the adoption of actually vicious procedures. These habits or methods, when systematized, we ordinarily refer to as principles, which we often believe in so conscientiously as to make of them a creed or dogma, and we are willing to concede they have the essentials of real fundamentals. As a matter of fact, few of our so-called scientific facts can be regarded as ultimately settled, at least to an extent that justifies the basing of methods of practice on an absolute rule of procedure that may not be changed for any reason.

Most of our activities, while capable of being considered as principles of practice, are only principles because some one has so labeled them, and by practice we have learned that their formulae usually work out that way, only they often enough don't, so that we speak of them with more or less skepticism. It is also very true that our sense of reason and experience entitle us to judgment, which enables us to assert ourselves when acting in the practical details of operative practice. This is paramount to saying, in view of our inability to dogmatize, that general sentiment and common practice should justify action when in doubt. There are usually enough unquestioned facts and theories to justify most of our methods of practice, but unfortunately at this time there are so many new as well as questioned traditional practices that there seems to be more or less disturbance as to what we should believe, and how we should act, in view of the present discussions as to what should be done with the diseased and threatened teeth.

To make a more or less pertinent observation, it would seem to us at this time that unless the present tendency to ill health from both local and systemic viewpoints are in some decisive way subverted, we are destined to be frightened to death or into expectant, if not into actual, neurosis. Some of us have already concluded we are destined to become either toothless, or woefully diseased at best. We can not, however, believe we are fated to become prematurely edentulous, nor hopelessly diseased. We have survived countless diseases more serious than dental caries and we have made scientific discoveries that have paved the way for the utilization of therapeutic remedies that we are confident will meet all our needs, when we know our dangers and the treatments that the present crisis calls for. We haven't as yet exhausted our resources in surgery and therapeutics, and we have as yet only begun to clear the territory in prophylaxis and preventive hygiene; why, then, should we be anxious as to the outcome of all the decades of resourceful surgical and conservative dental practice that has done so much to stabilize dentistry and make it a reputed and educated practice?

It would certainly be a serious blunder if all the talk and activity that is now centering about the pathology of focal infections of the teeth should result in changing our system of education and practice.

We have noted so many instances of what we believed were unjustifiable cases of extraction that we think it is quite time we were studying this question scientifically and clinically, so that we may as soon as practicable reach reliable conclusions as to procedures that are safe or advisable. In the meanwhile we can safely believe we are not justified when we are extracting ruthlessly, or even when there is only a possible cause which may justify a suspicion of probable harm. We certainly have not as yet reached that skill in diagnosis that will justify mandatory activity in all cases by the present methods we are using in diagnosis. We simply will have to adopt safer methods and secure more skillful operators before we can do either our patients or ourselves justice, in deciding on what operations can be wisely made, for the best interests of all concerned. The problem is so varied and the chances for gross errors are so liable that we need more careful study of this very important matter.

In the meantime, it will be the part of wisdom for us to keep a judicial mind, constantly on the watch for developments, ready to adopt always the approved practices when assured they are conservative and ethical. In a word, let us cultivate a scientific and ethical attitude, especially in all our professional activities which may not be definitely classified at this time. This will at least establish an equilibrium of thought and a balance in practice.

